# 6β-Chloro-5α-hydr­oxy-20-oxopregnan-3β-yl acetate

**DOI:** 10.1107/S1600536808019478

**Published:** 2008-07-05

**Authors:** Rui M. A. Pinto, Jorge A. R. Salvador, José A. Paixão, Ana Matos Beja, Manuela Ramos Silva

**Affiliations:** a Laboratório de Química Farmacêutica, Faculdade de Farmácia, Universidade de Coimbra, P-3000-295 Coimbra, Portugal; b CEMDRX, Departamento de Física, Faculdade de Ciências e Tecnologia, Universidade de Coimbra, P-3004-516, Coimbra, Portugal

## Abstract

The title steroid, C_23_H_35_ClO_4_, is a pregnane derivative prepared by ring opening of the corresponding 5α,6α-ep­oxy steroid with BiCl_3_. There are two symmetry-independent mol­ecules in the asymmetric unit that show no significant differences concerning bond lengths and angles. The conformation of the six-membered rings in both mol­ecules is close to a chair form, while the five-membered ring adopts an envelope conformation. All rings in both mol­ecules are *trans*-fused. The mol­ecules are held together by an extensive O—H⋯O hydrogen-bonding network.

## Related literature

For related literature, see: Pinto *et al.* (2007*a* ▶,*b* ▶,*c* ▶); Spickett *et al.* (2000 ▶); Mori *et al.* (1996 ▶), Iwashima *et al.* (2001 ▶), Dorta *et al.* (2004 ▶); Nittala *et al.* (1981 ▶); Cremer & Pople (1975 ▶).
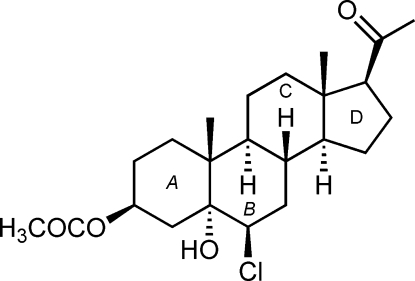


## Experimental

### 

#### Crystal data

C_23_H_35_ClO_4_
*M* *_r_* = 410.96
Monoclinic, 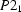

*a* = 7.6862 (6) Å
*b* = 27.484 (2) Å
*c* = 11.1863 (9) Å
β = 110.094 (2)°
*V* = 2219.2 (3) Å^3^
*Z* = 4
Mo *K*α radiation
μ = 0.20 mm^−1^
*T* = 293 (2) K
0.29 × 0.24 × 0.23 mm

#### Data collection

Bruker APEX CCD area-detector diffractometer
Absorption correction: multi-scan (*SADABS*; Sheldrick, 2000 ▶) *T*_min_ = 0.895, *T*_max_ = 0.956
55742 measured reflections
10978 independent reflections
7889 reflections with *I* > 2σ(*I*)
*R*_int_ = 0.032

#### Refinement

*R*[*F* ^2^ > 2σ(*F* ^2^)] = 0.042
*wR*(*F* ^2^) = 0.117
*S* = 1.00
10978 reflections
515 parameters
1 restraint
H-atom parameters constrained
Δρ_max_ = 0.22 e Å^−3^
Δρ_min_ = −0.26 e Å^−3^
Absolute structure: Flack (1983 ▶), 5188 Friedel pairs
Flack parameter: 0.04 (4)

### 

Data collection: *SMART* (Bruker, 2003 ▶); cell refinement: *SAINT* (Bruker, 2003 ▶); data reduction: *SAINT*; program(s) used to solve structure: *SHELXS97* (Sheldrick, 2008 ▶); program(s) used to refine structure: *SHELXL97 *(Sheldrick, 2008 ▶); molecular graphics: *PLATON* (Spek, 2003 ▶); software used to prepare material for publication: *SHELXL97*.

## Supplementary Material

Crystal structure: contains datablocks I, global. DOI: 10.1107/S1600536808019478/im2073sup1.cif

Structure factors: contains datablocks I. DOI: 10.1107/S1600536808019478/im2073Isup2.hkl

Additional supplementary materials:  crystallographic information; 3D view; checkCIF report

## Figures and Tables

**Table 1:** Hydrogen-bond geometry (Å, °)

*D*—H⋯*A*	*D*—H	H⋯*A*	*D*⋯*A*	*D*—H⋯*A*
O5*A*—H5*A*⋯O20*A*^i^	0.82	1.98	2.795 (2)	171
O5*B*—H5*B*⋯O20*B*^ii^	0.82	1.98	2.783 (2)	165

Symmetry codes: (i) 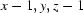; (ii) 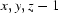.

**Fig. 1.**
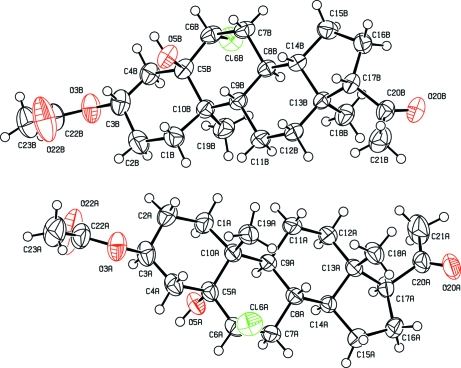
Molecular structure of I showing the atom numbering scheme of the two symmetry independent molecules (A and B). Displacement ellipsoids are drawn at the 50% level.

## supplementary crystallographic information

### Comment

In biological systems, hypochlorous acid, generated from H_2_O_2_ and chlorine by myeloperoxidase, is known to form chlorohydrin addition products with cholesterol (Spickett *et al.*, 2000). The vicinal chlorohydrin group has been identified in other steroid natural compounds, such as blattellastanoside B, an aggregation pheromone of German cockroach Blattella germanica (Mori *et al.*, 1996), marine steroids isolated from coral species (Iwashima *et al.*, 2001 and Dorta *et al.*, 2004) and chlorinated withanolides (Nittala *et al.*, 1981). As part of our current interest on epoxysteroid chemistry we recently reported new processes for the regioselective ring opening of epoxides with bismuth salts (Pinto *et al.*, 2007a). Applying this method to 5α,6α-epoxy-20-oxopregnan-3β-yl acetate we prepared the corresponding 6β-chloro-5α-hydroxysteroid (I) in 92% yield. The stereoselectivity of the nucleophilic ring opening of the 5α,6α-epoxide by BiCl_3_ was unequivocally demonstrated by this X-ray crystallographic study.

There are two symmetry independent molecules in the asymmetric unit (labeled A and B). In both molecules the conformation of the A ring is close to a chair form, as shown by the Cremer & Pople (1975) puckering parameters [A: Q= 0.570 (3) Å, θ = 5.8 (3)° and φ = 279 (3)°; B: Q= 0.578 (3) Å, θ = 4.9 (3)° and φ = 267 (3)°]. Rings B and C have slightly distorted chair conformations. The D-ring has a C13 envelope conformation with puckering parameters [A: q_2_ = 0.449 (2) Å and φ_2_ = 182.9 (3)°; B: q_2_ = 0.457 (2) Å and φ_2_ = 180.2 (3)°]. All rings in both molecules are fused *trans*. The acetoxy group at C3 is equatorial with respect to the A ring, and both substituents at the B ring are in axial position. The 17β-COCH_3_ substituent is equatorial with respect to the D-ring.

The molecules are hydrogen-bonded in infinite chains running parallel to [1 0 1] axis (molecule A) and [0 0 1] axis (molecule B) *via* the hydroxyl and the C20 carbonyl groups acting as donor and acceptor, respectively.

### Experimental

5α,6α-epoxysteroids were prepared from the corresponding Δ^5^-steroid by epoxidation with *m*-chloroperbenzoic acid. The synthesis of 6β-chloro-5α-hydroxy-20-oxopregnan-3β-yl acetate (I) was efficiently accomplished by ring opening of the corresponding 5α,6α-epoxysteroid with BiCl_3_ in 1,4-dioxane at 80°C. The product was isolated in 92% yield (Pinto *et al.*, 2007b). Recrystallization from methanol at room temperature gave colourless single crystals suitable for X-ray analysis. The analytical data of (I) are in accordance with published values (Pinto *et al.*, 2007c).

### Refinement

H atoms were positioned geometrically and refined using a riding model using *SHELXL97* default values (*U*_iso_(H) = 1.2 *U*_eq_(C) for CH and CH_2_ groups and Uĩso~(H) = 1.5 U~eq~(C) for CH_3_). The hydroxyl hydrogen atoms were refined with a distance restraint of 0.82 Å, starting from the difference map coordinates and with *U*_iso_(H) = 1.5 *U*_eq_(O).

### Crystal data

**Table:**

C_23_H_35_ClO_4_	*F*_000_ = 888
*M**_r_* = 410.96	*D*_x_ = 1.230 Mg m^−^^3^
Monoclinic, *P*2_1_	Melting point: 506 K
Hall symbol: P 2yb	Mo *K*α radiation λ = 0.71073 Å
*a* = 7.6862 (6) Å	Cell parameters from 9965 reflections
*b* = 27.484 (2) Å	θ = 2.4–26.1º
*c* = 11.1863 (9) Å	µ = 0.20 mm^−^^1^
β = 110.094 (2)º	*T* = 293 (2) K
*V* = 2219.2 (3) Å^3^	Prism, colourless
*Z* = 4	0.29 × 0.24 × 0.23 mm

### Data collection

**Table:**

Bruker APEX CCD area-detector diffractometer	10978 independent reflections
Radiation source: fine-focus sealed tube	7889 reflections with *I* > 2σ(*I*)
Monochromator: graphite	*R*_int_ = 0.032
*T* = 293(2) K	θ_max_ = 28.6º
φ and ω scans	θ_min_ = 1.9º
Absorption correction: multi-scan(SADABS; Sheldrick, 2000)	*h* = −10→10
*T*_min_ = 0.895, *T*_max_ = 0.956	*k* = −36→36
55742 measured reflections	*l* = −15→14

### Refinement

**Table:**

Refinement on *F*^2^	Hydrogen site location: inferred from neighbouring sites
Least-squares matrix: full	H-atom parameters constrained
*R*[*F*^2^ > 2σ(*F*^2^)] = 0.042	*w* = 1/[σ^2^(*F*_o_^2^) + (0.0626*P*)^2^ + 0.239*P*] where *P* = (*F*_o_^2^ + 2*F*_c_^2^)/3
*wR*(*F*^2^) = 0.117	(Δ/σ)_max_ < 0.001
*S* = 1.00	Δρ_max_ = 0.22 e Å^−^^3^
10978 reflections	Δρ_min_ = −0.26 e Å^−^^3^
515 parameters	Extinction correction: none
1 restraint	Absolute structure: Flack (1983), 5188 Friedel pairs
Primary atom site location: structure-invariant direct methods	Flack parameter: 0.04 (4)
Secondary atom site location: difference Fourier map	

### Special details

**Table:**

Geometry. All e.s.d.'s (except the e.s.d. in the dihedral angle between two l.s. planes) are estimated using the full covariance matrix. The cell e.s.d.'s are taken into account individually in the estimation of e.s.d.'s in distances, angles and torsion angles; correlations between e.s.d.'s in cell parameters are only used when they are defined by crystal symmetry. An approximate (isotropic) treatment of cell e.s.d.'s is used for estimating e.s.d.'s involving l.s. planes.
Refinement. Refinement of *F*^2^ against ALL reflections. The weighted *R*-factor *wR* and goodness of fit *S* are based on *F*^2^, conventional *R*-factors *R* are based on *F*, with *F* set to zero for negative *F*^2^. The threshold expression of *F*^2^ > σ(*F*^2^) is used only for calculating *R*-factors(gt) *etc*. and is not relevant to the choice of reflections for refinement. *R*-factors based on *F*^2^ are statistically about twice as large as those based on *F*, and *R*- factors based on ALL data will be even larger.

### Fractional atomic coordinates and isotropic or equivalent isotropic displacement parameters (Å^2^)

**Table:**

	*x*	*y*	*z*	*U*_iso_*/*U*_eq_	
C1A	0.4116 (3)	0.18353 (9)	0.7110 (2)	0.0504 (6)	
H1A1	0.5418	0.1913	0.7517	0.060*	
H1A2	0.4032	0.1502	0.6814	0.060*	
C2A	0.3296 (4)	0.21710 (10)	0.5962 (2)	0.0581 (6)	
H2A1	0.3524	0.2507	0.6235	0.070*	
H2A2	0.3903	0.2110	0.5347	0.070*	
C3A	0.1236 (4)	0.20907 (9)	0.5339 (2)	0.0538 (6)	
H3A	0.1008	0.1778	0.4893	0.065*	
C4A	0.0213 (3)	0.21092 (9)	0.6284 (2)	0.0508 (6)	
H4A1	−0.1075	0.2022	0.5854	0.061*	
H4A2	0.0250	0.2439	0.6603	0.061*	
C5A	0.1066 (3)	0.17630 (8)	0.7399 (2)	0.0412 (5)	
C6A	−0.0100 (3)	0.17224 (8)	0.8275 (2)	0.0460 (5)	
H6A	−0.1285	0.1576	0.7762	0.055*	
C7A	0.0739 (3)	0.13888 (8)	0.9396 (2)	0.0437 (5)	
H7A1	0.0614	0.1055	0.9098	0.052*	
H7A2	0.0058	0.1422	0.9978	0.052*	
C8A	0.2773 (3)	0.14963 (8)	1.01065 (19)	0.0374 (4)	
H8A	0.2872	0.1818	1.0500	0.045*	
C9A	0.3890 (3)	0.14992 (8)	0.91948 (19)	0.0378 (4)	
H9A	0.3719	0.1178	0.8791	0.045*	
C10A	0.3142 (3)	0.18762 (7)	0.81034 (19)	0.0388 (4)	
C11A	0.5973 (3)	0.15552 (10)	0.9918 (2)	0.0520 (6)	
H11A	0.6635	0.1506	0.9330	0.062*	
H11B	0.6218	0.1885	1.0241	0.062*	
C12A	0.6718 (3)	0.11993 (10)	1.1028 (2)	0.0490 (5)	
H12A	0.8004	0.1276	1.1499	0.059*	
H12B	0.6667	0.0871	1.0697	0.059*	
C13A	0.5605 (3)	0.12211 (8)	1.19318 (19)	0.0394 (4)	
C14A	0.3578 (3)	0.11236 (7)	1.11474 (19)	0.0363 (4)	
H14A	0.3566	0.0814	1.0710	0.044*	
C15A	0.2637 (3)	0.10218 (9)	1.2122 (2)	0.0468 (5)	
H15A	0.1610	0.0798	1.1777	0.056*	
H15B	0.2174	0.1321	1.2363	0.056*	
C16A	0.4139 (3)	0.07964 (9)	1.3267 (2)	0.0506 (5)	
H16A	0.3808	0.0467	1.3413	0.061*	
H16B	0.4311	0.0987	1.4029	0.061*	
C17A	0.5914 (3)	0.07993 (8)	1.29204 (19)	0.0430 (5)	
H17A	0.5965	0.0493	1.2486	0.052*	
C18A	0.5863 (4)	0.17114 (9)	1.2621 (2)	0.0546 (6)	
H18A	0.7137	0.1749	1.3150	0.082*	
H18B	0.5094	0.1723	1.3139	0.082*	
H18C	0.5519	0.1969	1.2005	0.082*	
C19A	0.3459 (3)	0.23976 (8)	0.8643 (2)	0.0502 (5)	
H19A	0.2662	0.2618	0.8034	0.075*	
H19B	0.4728	0.2489	0.8814	0.075*	
H19C	0.3185	0.2410	0.9418	0.075*	
C2OA	0.7669 (3)	0.08453 (9)	1.4041 (2)	0.0501 (6)	
C21A	0.9416 (4)	0.06847 (15)	1.3866 (3)	0.0835 (10)	
H21A	0.9664	0.0889	1.3247	0.125*	
H21B	0.9289	0.0353	1.3578	0.125*	
H21C	1.0422	0.0709	1.4662	0.125*	
C22A	0.0374 (4)	0.24333 (11)	0.3249 (3)	0.0637 (7)	
C23A	−0.0545 (5)	0.28581 (14)	0.2463 (3)	0.0882 (10)	
H23A	−0.1098	0.2759	0.1591	0.132*	
H23B	0.0357	0.3108	0.2526	0.132*	
H23C	−0.1487	0.2982	0.2764	0.132*	
Cl6A	−0.06419 (9)	0.23082 (2)	0.88169 (7)	0.06487 (18)	
O3A	0.0438 (3)	0.24819 (6)	0.44440 (16)	0.0661 (5)	
O5A	0.1045 (2)	0.12731 (5)	0.69162 (15)	0.0487 (4)	
H5A	0.0004	0.1209	0.6422	0.073*	
O20A	0.7689 (3)	0.10243 (8)	1.50390 (16)	0.0668 (5)	
O22A	0.0948 (5)	0.20927 (11)	0.2873 (2)	0.1296 (13)	
C1B	0.4187 (3)	0.40216 (10)	0.6303 (2)	0.0553 (6)	
H1B1	0.3966	0.4358	0.6028	0.066*	
H1B2	0.3309	0.3937	0.6718	0.066*	
C2B	0.3846 (4)	0.36956 (11)	0.5131 (2)	0.0642 (7)	
H2B1	0.3903	0.3357	0.5388	0.077*	
H2B2	0.2614	0.3758	0.4529	0.077*	
C3B	0.5266 (4)	0.37864 (9)	0.4488 (2)	0.0548 (6)	
H3B	0.5062	0.4109	0.4092	0.066*	
C4B	0.7206 (3)	0.37494 (9)	0.5377 (2)	0.0489 (5)	
H4B1	0.8052	0.3837	0.4938	0.059*	
H4B2	0.7468	0.3417	0.5677	0.059*	
C5B	0.7489 (3)	0.40931 (7)	0.6515 (2)	0.0422 (5)	
C6B	0.9517 (3)	0.41362 (8)	0.7339 (2)	0.0445 (5)	
H6B	1.0167	0.4278	0.6807	0.053*	
C7B	0.9786 (3)	0.44826 (8)	0.8449 (2)	0.0436 (5)	
H7B1	0.9552	0.4812	0.8125	0.052*	
H7B2	1.1065	0.4466	0.9013	0.052*	
C8B	0.8516 (3)	0.43712 (8)	0.9215 (2)	0.0370 (4)	
H8B	0.8857	0.4053	0.9623	0.044*	
C9B	0.6487 (3)	0.43539 (8)	0.8352 (2)	0.0397 (5)	
H9B	0.6202	0.4674	0.7943	0.048*	
C10B	0.6149 (3)	0.39743 (8)	0.7259 (2)	0.0417 (5)	
C11B	0.5181 (3)	0.42880 (10)	0.9126 (2)	0.0518 (6)	
H11C	0.3911	0.4324	0.8556	0.062*	
H11D	0.5320	0.3959	0.9462	0.062*	
C12B	0.5521 (3)	0.46465 (9)	1.0234 (2)	0.0465 (5)	
H12C	0.5187	0.4972	0.9902	0.056*	
H12D	0.4746	0.4560	1.0727	0.056*	
C13B	0.7535 (3)	0.46384 (7)	1.10859 (19)	0.0368 (4)	
C14B	0.8724 (3)	0.47500 (7)	1.02440 (19)	0.0366 (4)	
H14B	0.8241	0.5055	0.9799	0.044*	
C15B	1.0630 (3)	0.48659 (9)	1.1175 (2)	0.0467 (5)	
H15C	1.1246	0.5105	1.0822	0.056*	
H15D	1.1389	0.4575	1.1390	0.056*	
C16B	1.0279 (3)	0.50710 (9)	1.2361 (2)	0.0478 (5)	
H16C	1.0896	0.4872	1.3102	0.057*	
H16D	1.0745	0.5401	1.2532	0.057*	
C17B	0.8184 (3)	0.50615 (8)	1.20557 (19)	0.0406 (5)	
H17B	0.7672	0.5365	1.1614	0.049*	
C18B	0.8034 (4)	0.41460 (8)	1.1769 (2)	0.0503 (6)	
H18D	0.9320	0.4145	1.2293	0.075*	
H18E	0.7807	0.3890	1.1150	0.075*	
H18F	0.7289	0.4095	1.2293	0.075*	
C19B	0.6383 (4)	0.34516 (9)	0.7812 (2)	0.0549 (6)	
H19D	0.5293	0.3361	0.7993	0.082*	
H19E	0.7439	0.3441	0.8582	0.082*	
H19F	0.6562	0.3229	0.7204	0.082*	
C20B	0.7553 (3)	0.50139 (8)	1.3198 (2)	0.0471 (5)	
C21B	0.5746 (4)	0.52302 (12)	1.3101 (3)	0.0698 (8)	
H21D	0.4829	0.5138	1.2306	0.105*	
H21E	0.5855	0.5578	1.3143	0.105*	
H21F	0.5383	0.5115	1.3790	0.105*	
C22B	0.3880 (4)	0.34971 (11)	0.2356 (2)	0.0614 (7)	
C23B	0.3812 (5)	0.30944 (13)	0.1450 (3)	0.0813 (9)	
H23D	0.2673	0.3112	0.0740	0.122*	
H23E	0.3882	0.2788	0.1874	0.122*	
H23F	0.4839	0.3124	0.1151	0.122*	
Cl6B	1.05925 (9)	0.35550 (2)	0.79004 (6)	0.06012 (16)	
O3B	0.5069 (3)	0.34127 (6)	0.35021 (15)	0.0634 (5)	
O5B	0.7007 (2)	0.45778 (5)	0.60311 (15)	0.0505 (4)	
H5B	0.7621	0.4653	0.5588	0.076*	
O20B	0.8475 (3)	0.48017 (7)	1.41448 (15)	0.0627 (5)	
O22B	0.3020 (4)	0.38579 (10)	0.2084 (2)	0.1228 (12)	

### Atomic displacement parameters (Å^2^)

**Table:**

	*U*^11^	*U*^22^	*U*^33^	*U*^12^	*U*^13^	*U*^23^
C1A	0.0510 (14)	0.0574 (15)	0.0419 (12)	0.0034 (10)	0.0150 (11)	0.0095 (10)
C2A	0.0651 (16)	0.0623 (16)	0.0487 (13)	−0.0012 (12)	0.0217 (12)	0.0173 (12)
C3A	0.0678 (16)	0.0402 (12)	0.0417 (12)	0.0063 (11)	0.0038 (11)	0.0096 (10)
C4A	0.0481 (13)	0.0481 (13)	0.0439 (12)	0.0025 (10)	0.0000 (10)	0.0076 (10)
C5A	0.0440 (12)	0.0361 (11)	0.0366 (11)	−0.0005 (9)	0.0049 (9)	0.0040 (9)
C6A	0.0350 (11)	0.0461 (12)	0.0509 (13)	0.0010 (9)	0.0072 (10)	0.0009 (10)
C7A	0.0334 (10)	0.0491 (13)	0.0458 (12)	−0.0041 (9)	0.0101 (9)	0.0067 (10)
C8A	0.0324 (10)	0.0375 (11)	0.0386 (10)	−0.0017 (8)	0.0073 (8)	0.0023 (8)
C9A	0.0327 (10)	0.0417 (11)	0.0359 (10)	−0.0035 (8)	0.0079 (8)	0.0027 (9)
C10A	0.0402 (11)	0.0362 (11)	0.0362 (10)	−0.0038 (8)	0.0084 (9)	0.0034 (8)
C11A	0.0333 (11)	0.0733 (16)	0.0458 (12)	−0.0055 (11)	0.0091 (10)	0.0148 (12)
C12A	0.0332 (11)	0.0665 (15)	0.0431 (12)	0.0015 (10)	0.0077 (9)	0.0109 (11)
C13A	0.0355 (10)	0.0408 (11)	0.0368 (10)	−0.0050 (9)	0.0060 (9)	0.0015 (9)
C14A	0.0377 (10)	0.0349 (10)	0.0337 (10)	−0.0053 (8)	0.0088 (8)	0.0009 (8)
C15A	0.0477 (12)	0.0506 (13)	0.0435 (12)	−0.0040 (10)	0.0176 (10)	0.0046 (10)
C16A	0.0590 (14)	0.0536 (13)	0.0390 (12)	−0.0064 (11)	0.0167 (11)	0.0081 (10)
C17A	0.0498 (12)	0.0391 (11)	0.0326 (10)	−0.0016 (9)	0.0047 (9)	0.0013 (9)
C18A	0.0595 (15)	0.0458 (13)	0.0469 (13)	−0.0124 (11)	0.0032 (11)	−0.0014 (10)
C19A	0.0530 (13)	0.0447 (13)	0.0477 (12)	−0.0119 (10)	0.0105 (11)	0.0026 (10)
C2OA	0.0514 (14)	0.0510 (13)	0.0387 (12)	0.0009 (10)	0.0037 (10)	0.0109 (10)
C21A	0.0609 (18)	0.117 (3)	0.0589 (17)	0.0325 (18)	0.0034 (14)	0.0123 (17)
C22A	0.0743 (18)	0.0674 (18)	0.0447 (14)	0.0041 (14)	0.0144 (13)	0.0139 (13)
C23A	0.103 (3)	0.086 (2)	0.0648 (18)	0.0154 (19)	0.0161 (18)	0.0360 (17)
Cl6A	0.0620 (4)	0.0579 (4)	0.0788 (4)	0.0118 (3)	0.0295 (3)	0.0038 (3)
O3A	0.0924 (14)	0.0530 (11)	0.0446 (9)	0.0147 (9)	0.0126 (9)	0.0137 (8)
O5A	0.0499 (9)	0.0404 (8)	0.0420 (8)	−0.0033 (7)	−0.0018 (7)	−0.0026 (7)
O20A	0.0627 (11)	0.0850 (13)	0.0395 (9)	−0.0035 (10)	0.0006 (8)	−0.0078 (9)
O22A	0.224 (4)	0.110 (2)	0.0622 (14)	0.076 (2)	0.0589 (19)	0.0231 (14)
C1B	0.0473 (14)	0.0706 (16)	0.0489 (13)	−0.0045 (12)	0.0174 (11)	−0.0087 (12)
C2B	0.0616 (16)	0.0720 (18)	0.0508 (14)	−0.0077 (13)	0.0089 (13)	−0.0108 (13)
C3B	0.0785 (18)	0.0418 (12)	0.0449 (13)	0.0066 (11)	0.0221 (13)	−0.0081 (10)
C4B	0.0595 (15)	0.0458 (12)	0.0451 (12)	0.0028 (10)	0.0226 (11)	−0.0054 (10)
C5B	0.0565 (13)	0.0331 (11)	0.0426 (11)	0.0013 (9)	0.0242 (10)	−0.0004 (9)
C6B	0.0485 (13)	0.0427 (12)	0.0519 (13)	−0.0017 (9)	0.0296 (11)	−0.0033 (10)
C7B	0.0465 (12)	0.0420 (12)	0.0519 (13)	−0.0060 (9)	0.0290 (11)	−0.0067 (10)
C8B	0.0393 (11)	0.0377 (10)	0.0398 (11)	0.0008 (8)	0.0210 (9)	−0.0011 (8)
C9B	0.0401 (11)	0.0441 (11)	0.0390 (11)	−0.0026 (9)	0.0190 (9)	−0.0020 (9)
C10B	0.0455 (12)	0.0425 (12)	0.0398 (11)	−0.0039 (9)	0.0182 (10)	−0.0022 (9)
C11B	0.0466 (13)	0.0704 (16)	0.0444 (12)	−0.0124 (11)	0.0234 (11)	−0.0092 (11)
C12B	0.0395 (12)	0.0643 (15)	0.0417 (11)	−0.0011 (10)	0.0217 (10)	−0.0007 (10)
C13B	0.0416 (11)	0.0381 (11)	0.0352 (10)	−0.0018 (9)	0.0189 (9)	−0.0001 (8)
C14B	0.0389 (11)	0.0349 (10)	0.0397 (10)	0.0002 (8)	0.0184 (9)	0.0025 (8)
C15B	0.0403 (12)	0.0485 (13)	0.0523 (13)	−0.0028 (10)	0.0172 (10)	−0.0068 (10)
C16B	0.0505 (13)	0.0497 (13)	0.0398 (11)	−0.0035 (10)	0.0113 (10)	−0.0023 (10)
C17B	0.0515 (12)	0.0384 (11)	0.0351 (10)	0.0024 (9)	0.0189 (9)	0.0030 (8)
C18B	0.0686 (16)	0.0396 (12)	0.0452 (12)	−0.0035 (11)	0.0229 (12)	0.0049 (10)
C19B	0.0709 (16)	0.0452 (13)	0.0539 (14)	−0.0161 (11)	0.0281 (13)	−0.0035 (10)
C20B	0.0641 (15)	0.0418 (12)	0.0395 (12)	−0.0050 (10)	0.0230 (11)	−0.0064 (10)
C21B	0.0766 (19)	0.086 (2)	0.0576 (15)	0.0125 (15)	0.0374 (15)	−0.0009 (14)
C22B	0.0684 (16)	0.0633 (17)	0.0448 (13)	0.0109 (14)	0.0097 (12)	−0.0091 (12)
C23B	0.091 (2)	0.083 (2)	0.0593 (17)	0.0097 (18)	0.0113 (16)	−0.0246 (16)
Cl6B	0.0586 (4)	0.0533 (3)	0.0693 (4)	0.0115 (3)	0.0231 (3)	−0.0046 (3)
O3B	0.0874 (13)	0.0495 (10)	0.0435 (9)	0.0127 (9)	0.0102 (9)	−0.0098 (7)
O5B	0.0747 (11)	0.0405 (8)	0.0456 (9)	0.0064 (8)	0.0327 (8)	0.0044 (7)
O20B	0.0801 (13)	0.0724 (12)	0.0388 (9)	0.0022 (10)	0.0244 (9)	0.0072 (8)
O22B	0.154 (3)	0.0963 (18)	0.0704 (14)	0.0630 (19)	−0.0223 (16)	−0.0202 (13)

### Geometric parameters (Å, °)

**Table:**

C1A—C2A	1.530 (3)	C1B—C10B	1.525 (3)
C1A—C10A	1.542 (3)	C1B—C2B	1.534 (3)
C1A—H1A1	0.9700	C1B—H1B1	0.9700
C1A—H1A2	0.9700	C1B—H1B2	0.9700
C2A—C3A	1.510 (4)	C2B—C3B	1.521 (4)
C2A—H2A1	0.9700	C2B—H2B1	0.9700
C2A—H2A2	0.9700	C2B—H2B2	0.9700
C3A—O3A	1.454 (3)	C3B—O3B	1.476 (3)
C3A—C4A	1.520 (4)	C3B—C4B	1.484 (4)
C3A—H3A	0.9800	C3B—H3B	0.9800
C4A—C5A	1.526 (3)	C4B—C5B	1.539 (3)
C4A—H4A1	0.9700	C4B—H4B1	0.9700
C4A—H4A2	0.9700	C4B—H4B2	0.9700
C5A—O5A	1.449 (3)	C5B—O5B	1.438 (3)
C5A—C6A	1.542 (3)	C5B—C6B	1.518 (3)
C5A—C10A	1.549 (3)	C5B—C10B	1.566 (3)
C6A—C7A	1.508 (3)	C6B—C7B	1.521 (3)
C6A—Cl6A	1.819 (2)	C6B—Cl6B	1.809 (2)
C6A—H6A	0.9800	C6B—H6B	0.9800
C7A—C8A	1.520 (3)	C7B—C8B	1.535 (3)
C7A—H7A1	0.9700	C7B—H7B1	0.9700
C7A—H7A2	0.9700	C7B—H7B2	0.9700
C8A—C14A	1.514 (3)	C8B—C14B	1.519 (3)
C8A—C9A	1.542 (3)	C8B—C9B	1.527 (3)
C8A—H8A	0.9800	C8B—H8B	0.9800
C9A—C11A	1.534 (3)	C9B—C11B	1.545 (3)
C9A—C10A	1.553 (3)	C9B—C10B	1.560 (3)
C9A—H9A	0.9800	C9B—H9B	0.9800
C10A—C19A	1.542 (3)	C10B—C19B	1.550 (3)
C11A—C12A	1.529 (3)	C11B—C12B	1.534 (3)
C11A—H11A	0.9700	C11B—H11C	0.9700
C11A—H11B	0.9700	C11B—H11D	0.9700
C12A—C13A	1.533 (3)	C12B—C13B	1.513 (3)
C12A—H12A	0.9700	C12B—H12C	0.9700
C12A—H12B	0.9700	C12B—H12D	0.9700
C13A—C14A	1.527 (3)	C13B—C18B	1.537 (3)
C13A—C18A	1.531 (3)	C13B—C17B	1.551 (3)
C13A—C17A	1.563 (3)	C13B—C14B	1.551 (3)
C14A—C15A	1.527 (3)	C14B—C15B	1.512 (3)
C14A—H14A	0.9800	C14B—H14B	0.9800
C15A—C16A	1.530 (3)	C15B—C16B	1.548 (3)
C15A—H15A	0.9700	C15B—H15C	0.9700
C15A—H15B	0.9700	C15B—H15D	0.9700
C16A—C17A	1.541 (3)	C16B—C17B	1.527 (3)
C16A—H16A	0.9700	C16B—H16C	0.9700
C16A—H16B	0.9700	C16B—H16D	0.9700
C17A—C2OA	1.498 (3)	C17B—C20B	1.520 (3)
C17A—H17A	0.9800	C17B—H17B	0.9800
C18A—H18A	0.9600	C18B—H18D	0.9600
C18A—H18B	0.9600	C18B—H18E	0.9600
C18A—H18C	0.9600	C18B—H18F	0.9600
C19A—H19A	0.9600	C19B—H19D	0.9600
C19A—H19B	0.9600	C19B—H19E	0.9600
C19A—H19C	0.9600	C19B—H19F	0.9600
C2OA—O20A	1.215 (3)	C20B—O20B	1.204 (3)
C2OA—C21A	1.489 (4)	C20B—C21B	1.480 (4)
C21A—H21A	0.9600	C21B—H21D	0.9600
C21A—H21B	0.9600	C21B—H21E	0.9600
C21A—H21C	0.9600	C21B—H21F	0.9600
C22A—O22A	1.173 (4)	C22B—O22B	1.173 (4)
C22A—O3A	1.327 (3)	C22B—O3B	1.315 (3)
C22A—C23A	1.486 (4)	C22B—C23B	1.490 (4)
C23A—H23A	0.9600	C23B—H23D	0.9600
C23A—H23B	0.9600	C23B—H23E	0.9600
C23A—H23C	0.9600	C23B—H23F	0.9600
O5A—H5A	0.8200	O5B—H5B	0.8200
			
C2A—C1A—C10A	113.5 (2)	C10B—C1B—C2B	112.8 (2)
C2A—C1A—H1A1	108.9	C10B—C1B—H1B1	109.0
C10A—C1A—H1A1	108.9	C2B—C1B—H1B1	109.0
C2A—C1A—H1A2	108.9	C10B—C1B—H1B2	109.0
C10A—C1A—H1A2	108.9	C2B—C1B—H1B2	109.0
H1A1—C1A—H1A2	107.7	H1B1—C1B—H1B2	107.8
C3A—C2A—C1A	111.3 (2)	C3B—C2B—C1B	111.6 (2)
C3A—C2A—H2A1	109.4	C3B—C2B—H2B1	109.3
C1A—C2A—H2A1	109.4	C1B—C2B—H2B1	109.3
C3A—C2A—H2A2	109.4	C3B—C2B—H2B2	109.3
C1A—C2A—H2A2	109.4	C1B—C2B—H2B2	109.3
H2A1—C2A—H2A2	108.0	H2B1—C2B—H2B2	108.0
O3A—C3A—C2A	109.7 (2)	O3B—C3B—C4B	105.94 (19)
O3A—C3A—C4A	104.9 (2)	O3B—C3B—C2B	109.5 (2)
C2A—C3A—C4A	112.7 (2)	C4B—C3B—C2B	113.0 (2)
O3A—C3A—H3A	109.8	O3B—C3B—H3B	109.4
C2A—C3A—H3A	109.8	C4B—C3B—H3B	109.4
C4A—C3A—H3A	109.8	C2B—C3B—H3B	109.4
C3A—C4A—C5A	111.55 (19)	C3B—C4B—C5B	109.63 (18)
C3A—C4A—H4A1	109.3	C3B—C4B—H4B1	109.7
C5A—C4A—H4A1	109.3	C5B—C4B—H4B1	109.7
C3A—C4A—H4A2	109.3	C3B—C4B—H4B2	109.7
C5A—C4A—H4A2	109.3	C5B—C4B—H4B2	109.7
H4A1—C4A—H4A2	108.0	H4B1—C4B—H4B2	108.2
O5A—C5A—C4A	109.18 (17)	O5B—C5B—C6B	103.56 (17)
O5A—C5A—C6A	103.59 (17)	O5B—C5B—C4B	108.22 (17)
C4A—C5A—C6A	112.58 (18)	C6B—C5B—C4B	111.97 (18)
O5A—C5A—C10A	104.70 (16)	O5B—C5B—C10B	105.17 (16)
C4A—C5A—C10A	111.65 (17)	C6B—C5B—C10B	114.75 (17)
C6A—C5A—C10A	114.38 (17)	C4B—C5B—C10B	112.33 (18)
C7A—C6A—C5A	113.38 (18)	C5B—C6B—C7B	111.59 (17)
C7A—C6A—Cl6A	110.03 (16)	C5B—C6B—Cl6B	113.16 (15)
C5A—C6A—Cl6A	113.42 (16)	C7B—C6B—Cl6B	110.58 (16)
C7A—C6A—H6A	106.5	C5B—C6B—H6B	107.1
C5A—C6A—H6A	106.5	C7B—C6B—H6B	107.1
Cl6A—C6A—H6A	106.5	Cl6B—C6B—H6B	107.1
C6A—C7A—C8A	112.70 (17)	C6B—C7B—C8B	113.37 (17)
C6A—C7A—H7A1	109.1	C6B—C7B—H7B1	108.9
C8A—C7A—H7A1	109.1	C8B—C7B—H7B1	108.9
C6A—C7A—H7A2	109.1	C6B—C7B—H7B2	108.9
C8A—C7A—H7A2	109.1	C8B—C7B—H7B2	108.9
H7A1—C7A—H7A2	107.8	H7B1—C7B—H7B2	107.7
C14A—C8A—C7A	110.23 (16)	C14B—C8B—C9B	108.60 (16)
C14A—C8A—C9A	109.80 (16)	C14B—C8B—C7B	111.40 (17)
C7A—C8A—C9A	111.18 (16)	C9B—C8B—C7B	111.14 (16)
C14A—C8A—H8A	108.5	C14B—C8B—H8B	108.5
C7A—C8A—H8A	108.5	C9B—C8B—H8B	108.5
C9A—C8A—H8A	108.5	C7B—C8B—H8B	108.5
C11A—C9A—C8A	111.66 (17)	C8B—C9B—C11B	111.64 (16)
C11A—C9A—C10A	112.65 (17)	C8B—C9B—C10B	112.05 (17)
C8A—C9A—C10A	112.55 (16)	C11B—C9B—C10B	113.30 (18)
C11A—C9A—H9A	106.5	C8B—C9B—H9B	106.4
C8A—C9A—H9A	106.5	C11B—C9B—H9B	106.4
C10A—C9A—H9A	106.5	C10B—C9B—H9B	106.4
C1A—C10A—C19A	107.42 (18)	C1B—C10B—C19B	108.07 (19)
C1A—C10A—C5A	106.78 (17)	C1B—C10B—C9B	110.56 (18)
C19A—C10A—C5A	112.49 (18)	C19B—C10B—C9B	109.98 (17)
C1A—C10A—C9A	112.19 (17)	C1B—C10B—C5B	106.37 (17)
C19A—C10A—C9A	110.26 (17)	C19B—C10B—C5B	113.47 (18)
C5A—C10A—C9A	107.72 (16)	C9B—C10B—C5B	108.34 (17)
C12A—C11A—C9A	113.32 (18)	C12B—C11B—C9B	114.46 (19)
C12A—C11A—H11A	108.9	C12B—C11B—H11C	108.6
C9A—C11A—H11A	108.9	C9B—C11B—H11C	108.6
C12A—C11A—H11B	108.9	C12B—C11B—H11D	108.6
C9A—C11A—H11B	108.9	C9B—C11B—H11D	108.6
H11A—C11A—H11B	107.7	H11C—C11B—H11D	107.6
C11A—C12A—C13A	111.88 (19)	C13B—C12B—C11B	110.67 (18)
C11A—C12A—H12A	109.2	C13B—C12B—H12C	109.5
C13A—C12A—H12A	109.2	C11B—C12B—H12C	109.5
C11A—C12A—H12B	109.2	C13B—C12B—H12D	109.5
C13A—C12A—H12B	109.2	C11B—C12B—H12D	109.5
H12A—C12A—H12B	107.9	H12C—C12B—H12D	108.1
C14A—C13A—C12A	107.73 (16)	C12B—C13B—C18B	110.66 (18)
C14A—C13A—C18A	111.95 (18)	C12B—C13B—C17B	116.29 (17)
C12A—C13A—C18A	111.19 (19)	C18B—C13B—C17B	110.29 (17)
C14A—C13A—C17A	98.83 (16)	C12B—C13B—C14B	107.65 (16)
C12A—C13A—C17A	116.99 (18)	C18B—C13B—C14B	112.18 (17)
C18A—C13A—C17A	109.56 (17)	C17B—C13B—C14B	99.29 (15)
C8A—C14A—C15A	120.28 (18)	C15B—C14B—C8B	119.41 (17)
C8A—C14A—C13A	112.94 (16)	C15B—C14B—C13B	104.89 (16)
C15A—C14A—C13A	105.21 (16)	C8B—C14B—C13B	113.20 (17)
C8A—C14A—H14A	105.8	C15B—C14B—H14B	106.1
C15A—C14A—H14A	105.8	C8B—C14B—H14B	106.1
C13A—C14A—H14A	105.8	C13B—C14B—H14B	106.1
C14A—C15A—C16A	105.40 (18)	C14B—C15B—C16B	104.71 (17)
C14A—C15A—H15A	110.7	C14B—C15B—H15C	110.8
C16A—C15A—H15A	110.7	C16B—C15B—H15C	110.8
C14A—C15A—H15B	110.7	C14B—C15B—H15D	110.8
C16A—C15A—H15B	110.7	C16B—C15B—H15D	110.8
H15A—C15A—H15B	108.8	H15C—C15B—H15D	108.9
C15A—C16A—C17A	105.41 (17)	C17B—C16B—C15B	106.46 (17)
C15A—C16A—H16A	110.7	C17B—C16B—H16C	110.4
C17A—C16A—H16A	110.7	C15B—C16B—H16C	110.4
C15A—C16A—H16B	110.7	C17B—C16B—H16D	110.4
C17A—C16A—H16B	110.7	C15B—C16B—H16D	110.4
H16A—C16A—H16B	108.8	H16C—C16B—H16D	108.6
C2OA—C17A—C16A	114.21 (18)	C20B—C17B—C16B	115.49 (19)
C2OA—C17A—C13A	113.82 (18)	C20B—C17B—C13B	113.85 (17)
C16A—C17A—C13A	104.44 (17)	C16B—C17B—C13B	103.31 (17)
C2OA—C17A—H17A	108.0	C20B—C17B—H17B	107.9
C16A—C17A—H17A	108.0	C16B—C17B—H17B	107.9
C13A—C17A—H17A	108.0	C13B—C17B—H17B	107.9
C13A—C18A—H18A	109.5	C13B—C18B—H18D	109.5
C13A—C18A—H18B	109.5	C13B—C18B—H18E	109.5
H18A—C18A—H18B	109.5	H18D—C18B—H18E	109.5
C13A—C18A—H18C	109.5	C13B—C18B—H18F	109.5
H18A—C18A—H18C	109.5	H18D—C18B—H18F	109.5
H18B—C18A—H18C	109.5	H18E—C18B—H18F	109.5
C10A—C19A—H19A	109.5	C10B—C19B—H19D	109.5
C10A—C19A—H19B	109.5	C10B—C19B—H19E	109.5
H19A—C19A—H19B	109.5	H19D—C19B—H19E	109.5
C10A—C19A—H19C	109.5	C10B—C19B—H19F	109.5
H19A—C19A—H19C	109.5	H19D—C19B—H19F	109.5
H19B—C19A—H19C	109.5	H19E—C19B—H19F	109.5
O20A—C2OA—C21A	120.9 (2)	O20B—C20B—C21B	120.9 (2)
O20A—C2OA—C17A	121.8 (2)	O20B—C20B—C17B	121.3 (2)
C21A—C2OA—C17A	117.3 (2)	C21B—C20B—C17B	117.8 (2)
C2OA—C21A—H21A	109.5	C20B—C21B—H21D	109.5
C2OA—C21A—H21B	109.5	C20B—C21B—H21E	109.5
H21A—C21A—H21B	109.5	H21D—C21B—H21E	109.5
C2OA—C21A—H21C	109.5	C20B—C21B—H21F	109.5
H21A—C21A—H21C	109.5	H21D—C21B—H21F	109.5
H21B—C21A—H21C	109.5	H21E—C21B—H21F	109.5
O22A—C22A—O3A	123.6 (3)	O22B—C22B—O3B	122.9 (2)
O22A—C22A—C23A	125.1 (3)	O22B—C22B—C23B	124.6 (3)
O3A—C22A—C23A	111.2 (3)	O3B—C22B—C23B	112.5 (2)
C22A—C23A—H23A	109.5	C22B—C23B—H23D	109.5
C22A—C23A—H23B	109.5	C22B—C23B—H23E	109.5
H23A—C23A—H23B	109.5	H23D—C23B—H23E	109.5
C22A—C23A—H23C	109.5	C22B—C23B—H23F	109.5
H23A—C23A—H23C	109.5	H23D—C23B—H23F	109.5
H23B—C23A—H23C	109.5	H23E—C23B—H23F	109.5
C22A—O3A—C3A	118.7 (2)	C22B—O3B—C3B	117.69 (19)
C5A—O5A—H5A	109.5	C5B—O5B—H5B	109.5
			
C10A—C1A—C2A—C3A	−54.8 (3)	C10B—C1B—C2B—C3B	−54.8 (3)
C1A—C2A—C3A—O3A	167.5 (2)	C1B—C2B—C3B—O3B	170.9 (2)
C1A—C2A—C3A—C4A	51.0 (3)	C1B—C2B—C3B—C4B	53.1 (3)
O3A—C3A—C4A—C5A	−172.38 (18)	O3B—C3B—C4B—C5B	−174.52 (18)
C2A—C3A—C4A—C5A	−53.1 (3)	C2B—C3B—C4B—C5B	−54.7 (3)
C3A—C4A—C5A—O5A	−57.7 (2)	C3B—C4B—C5B—O5B	−56.8 (2)
C3A—C4A—C5A—C6A	−172.21 (19)	C3B—C4B—C5B—C6B	−170.28 (19)
C3A—C4A—C5A—C10A	57.5 (2)	C3B—C4B—C5B—C10B	58.9 (2)
O5A—C5A—C6A—C7A	63.2 (2)	O5B—C5B—C6B—C7B	62.7 (2)
C4A—C5A—C6A—C7A	−178.95 (19)	C4B—C5B—C6B—C7B	179.12 (17)
C10A—C5A—C6A—C7A	−50.1 (2)	C10B—C5B—C6B—C7B	−51.3 (2)
O5A—C5A—C6A—Cl6A	−170.33 (14)	O5B—C5B—C6B—Cl6B	−171.77 (12)
C4A—C5A—C6A—Cl6A	−52.5 (2)	C4B—C5B—C6B—Cl6B	−55.4 (2)
C10A—C5A—C6A—Cl6A	76.3 (2)	C10B—C5B—C6B—Cl6B	74.2 (2)
C5A—C6A—C7A—C8A	49.3 (3)	C5B—C6B—C7B—C8B	50.8 (2)
Cl6A—C6A—C7A—C8A	−78.9 (2)	Cl6B—C6B—C7B—C8B	−76.1 (2)
C6A—C7A—C8A—C14A	−175.11 (18)	C6B—C7B—C8B—C14B	−175.41 (19)
C6A—C7A—C8A—C9A	−53.1 (2)	C6B—C7B—C8B—C9B	−54.2 (2)
C14A—C8A—C9A—C11A	−51.9 (2)	C14B—C8B—C9B—C11B	−52.0 (2)
C7A—C8A—C9A—C11A	−174.15 (19)	C7B—C8B—C9B—C11B	−174.88 (18)
C14A—C8A—C9A—C10A	−179.76 (17)	C14B—C8B—C9B—C10B	179.71 (17)
C7A—C8A—C9A—C10A	58.0 (2)	C7B—C8B—C9B—C10B	56.8 (2)
C2A—C1A—C10A—C19A	−63.7 (3)	C2B—C1B—C10B—C19B	−65.9 (3)
C2A—C1A—C10A—C5A	57.1 (2)	C2B—C1B—C10B—C9B	173.7 (2)
C2A—C1A—C10A—C9A	174.93 (19)	C2B—C1B—C10B—C5B	56.3 (3)
O5A—C5A—C10A—C1A	60.0 (2)	C8B—C9B—C10B—C1B	−171.23 (18)
C4A—C5A—C10A—C1A	−58.0 (2)	C11B—C9B—C10B—C1B	61.4 (2)
C6A—C5A—C10A—C1A	172.66 (18)	C8B—C9B—C10B—C19B	69.5 (2)
O5A—C5A—C10A—C19A	177.57 (17)	C11B—C9B—C10B—C19B	−57.9 (2)
C4A—C5A—C10A—C19A	59.6 (2)	C8B—C9B—C10B—C5B	−55.0 (2)
C6A—C5A—C10A—C19A	−69.8 (2)	C11B—C9B—C10B—C5B	177.55 (19)
O5A—C5A—C10A—C9A	−60.7 (2)	O5B—C5B—C10B—C1B	58.7 (2)
C4A—C5A—C10A—C9A	−178.72 (18)	C6B—C5B—C10B—C1B	171.78 (18)
C6A—C5A—C10A—C9A	52.0 (2)	C4B—C5B—C10B—C1B	−58.8 (2)
C11A—C9A—C10A—C1A	59.4 (2)	O5B—C5B—C10B—C19B	177.35 (18)
C8A—C9A—C10A—C1A	−173.28 (17)	C6B—C5B—C10B—C19B	−69.5 (2)
C11A—C9A—C10A—C19A	−60.3 (2)	C4B—C5B—C10B—C19B	59.9 (2)
C8A—C9A—C10A—C19A	67.0 (2)	O5B—C5B—C10B—C9B	−60.2 (2)
C11A—C9A—C10A—C5A	176.62 (19)	C6B—C5B—C10B—C9B	52.9 (2)
C8A—C9A—C10A—C5A	−56.1 (2)	C4B—C5B—C10B—C9B	−177.70 (18)
C8A—C9A—C11A—C12A	50.0 (3)	C8B—C9B—C11B—C12B	50.6 (3)
C10A—C9A—C11A—C12A	177.81 (19)	C10B—C9B—C11B—C12B	178.2 (2)
C9A—C11A—C12A—C13A	−52.9 (3)	C9B—C11B—C12B—C13B	−53.0 (3)
C11A—C12A—C13A—C14A	56.2 (2)	C11B—C12B—C13B—C18B	−66.9 (2)
C11A—C12A—C13A—C18A	−66.8 (2)	C11B—C12B—C13B—C17B	166.29 (18)
C11A—C12A—C13A—C17A	166.34 (18)	C11B—C12B—C13B—C14B	56.0 (2)
C7A—C8A—C14A—C15A	−52.7 (3)	C9B—C8B—C14B—C15B	−175.70 (17)
C9A—C8A—C14A—C15A	−175.50 (18)	C7B—C8B—C14B—C15B	−53.0 (2)
C7A—C8A—C14A—C13A	−177.93 (17)	C9B—C8B—C14B—C13B	60.1 (2)
C9A—C8A—C14A—C13A	59.3 (2)	C7B—C8B—C14B—C13B	−177.21 (18)
C12A—C13A—C14A—C8A	−60.9 (2)	C12B—C13B—C14B—C15B	165.70 (18)
C18A—C13A—C14A—C8A	61.6 (2)	C18B—C13B—C14B—C15B	−72.3 (2)
C17A—C13A—C14A—C8A	176.94 (17)	C17B—C13B—C14B—C15B	44.17 (19)
C12A—C13A—C14A—C15A	166.02 (18)	C12B—C13B—C14B—C8B	−62.5 (2)
C18A—C13A—C14A—C15A	−71.4 (2)	C18B—C13B—C14B—C8B	59.5 (2)
C17A—C13A—C14A—C15A	43.9 (2)	C17B—C13B—C14B—C8B	176.00 (16)
C8A—C14A—C15A—C16A	−158.59 (19)	C8B—C14B—C15B—C16B	−156.10 (19)
C13A—C14A—C15A—C16A	−29.8 (2)	C13B—C14B—C15B—C16B	−27.9 (2)
C14A—C15A—C16A—C17A	2.4 (2)	C14B—C15B—C16B—C17B	0.2 (2)
C15A—C16A—C17A—C2OA	149.9 (2)	C15B—C16B—C17B—C20B	152.35 (19)
C15A—C16A—C17A—C13A	25.0 (2)	C15B—C16B—C17B—C13B	27.4 (2)
C14A—C13A—C17A—C2OA	−167.17 (19)	C12B—C13B—C17B—C20B	75.8 (2)
C12A—C13A—C17A—C2OA	77.7 (2)	C18B—C13B—C17B—C20B	−51.3 (2)
C18A—C13A—C17A—C2OA	−50.0 (3)	C14B—C13B—C17B—C20B	−169.18 (18)
C14A—C13A—C17A—C16A	−42.0 (2)	C12B—C13B—C17B—C16B	−158.21 (17)
C12A—C13A—C17A—C16A	−157.10 (19)	C18B—C13B—C17B—C16B	74.8 (2)
C18A—C13A—C17A—C16A	75.2 (2)	C14B—C13B—C17B—C16B	−43.16 (19)
C16A—C17A—C2OA—O20A	−23.3 (3)	C16B—C17B—C20B—O20B	−29.8 (3)
C13A—C17A—C2OA—O20A	96.5 (3)	C13B—C17B—C20B—O20B	89.5 (3)
C16A—C17A—C2OA—C21A	159.6 (3)	C16B—C17B—C20B—C21B	151.2 (2)
C13A—C17A—C2OA—C21A	−80.6 (3)	C13B—C17B—C20B—C21B	−89.5 (3)
O22A—C22A—O3A—C3A	−0.9 (5)	O22B—C22B—O3B—C3B	2.0 (5)
C23A—C22A—O3A—C3A	177.9 (3)	C23B—C22B—O3B—C3B	179.9 (3)
C2A—C3A—O3A—C22A	89.1 (3)	C4B—C3B—O3B—C22B	−150.3 (2)
C4A—C3A—O3A—C22A	−149.6 (2)	C2B—C3B—O3B—C22B	87.5 (3)

### Hydrogen-bond geometry (Å, °)

**Table:**

*D*—H···*A*	*D*—H	H···*A*	*D*···*A*	*D*—H···*A*
O5A—H5A···O20A^i^	0.82	1.98	2.795 (2)	171
O5B—H5B···O20B^ii^	0.82	1.98	2.783 (2)	165

Symmetry codes: (i) *x*−1, *y*, *z*−1; (ii) *x*, *y*, *z*−1.

## References

[bb1] Bruker (2003). *SMART* and *SAINT* Bruker AXS Inc., Madison, Wisconsin, USA.

[bb2] Cremer, D. & Pople, J. A. (1975). *J. Am. Chem. Soc.* **97**, 1354–1358.

[bb3] Dorta, E., Díaz-Marrero, A. R., Cueto, M., D’Croz, L., Maté, J. L., San-Martín, A. & Darias, J. (2004). *Tetrahedron Lett.* **45**, 915–918.

[bb4] Flack, H. D. (1983). *Acta Cryst.* A**39**, 876–881.

[bb5] Iwashima, M., Nara, K., Nakamichi, Y. & Iguchi, K. (2001). *Steroids*, **66**, 25–32. 10.1016/s0039-128x(00)00144-6 11090655

[bb6] Mori, K., Nakayama, T. & Sakuma, M. (1996). *Bioorg. Med. Chem.* **4**, 401–408. 10.1016/0968-0896(96)00018-1 8733618

[bb7] Nittala, S. S., Velde, V. V., Frolow, F. & Lavie, D. (1981). *Phytochemistry*, **20**, 2547–2552.

[bb8] Pinto, R. M. A., Ramos Silva, M., Matos Beja, A. & Salvador, J. A. R. (2007*a*). *Acta Cryst.* E**63**, o2138–o2139.

[bb9] Pinto, R. M. A., Ramos Silva, M., Matos Beja, A., Salvador, J. A. R. & Paixão, J. A. (2007*b*). *Acta Cryst.* E**63**, o3321.

[bb10] Pinto, R. M. A., Salvador, J. A. R. & Le Roux, C. (2007*c*). *Tetrahedron*, **63**, 9221–9228.

[bb11] Sheldrick, G. M. (2000). *SADABS* University of Göttingen, Germany.

[bb12] Sheldrick, G. M. (2008). *Acta Cryst.* A**64**, 112–122. 10.1107/S0108767307043930 18156677

[bb13] Spek, A. L. (2003). *J. Appl. Cryst.* **36**, 7–13.

[bb14] Spickett, C. M., Jerlich, A., Panasenko, O. M., Arnhold, J., Pitt, A. R., Stelmaszynska, T. & Schaur, R. J. (2000). *Acta Biochim. Pol.* **47**, 889–897. 11996112

